# Candida Parapsilosis: a Rare Culprit of Shunt Infection in an Adult

**DOI:** 10.1155/cris/6687581

**Published:** 2025-04-15

**Authors:** Ersin Ikizoglu, Mert Arslan, Irmak Guzel, Ceren Kizmazoglu, Vildan Avkan Oguz, Burak Sade

**Affiliations:** ^1^Department of Neurosurgery, Dokuz Eylul University School of Medicine, Izmir, Türkiye; ^2^Department of Medical Microbiology, Dokuz Eylul University School of Medicine, Izmir, Türkiye; ^3^Department of Infectious Diseases and Clinical Microbiology, Dokuz Eylul University School of Medicine, Izmir, Türkiye

## Abstract

*Candida parapsilosis* is an exceedingly rare cause of ventriculoperitoneal (VP) shunt infection, even in patients who have a history of long-term antibiotic use, immune-compromised newborns, and intensive care unit patients. We hereby report a case of a 53-year-old male who presented with subarachnoid hemorrhage and had a complicated postoperative course due to *C. parapsilosis* infection, and we discuss the pertinent clinical aspects.

## 1. Introduction

Infection is a dreaded complication of shunt surgery [1]. While bacterial skin flora is commonly encountered in shunt infections, fungal culprits are quite rare [1, 2]. We hereby present a case with shunt infection due to *Candida parapsilosis*.

## 2. Case Report

A 53-year-old male presented with a sudden onset of confusion and collapse. His past medical history was significant for hypertension. Neurological exam showed a Glasgow Coma Score of 10 with no lateralized motor signs. Computed tomography (CT) revealed Fisher 4 subarachnoid hemorrhage with hydrocephalus. An external ventricular drain (EVD) was placed under ampicillin sulbactam prophylaxis. CT angiogram and digital subtraction angiography showed a dissecting aneurysm of the right vertebral artery. This was treated with coil embolization, during which the right posterior inferior cerebellar artery (PICA) had to be sacrificed. Follow-up CT showed acute infarction and increased edema in the right PICA territory, with compression of the fourth ventricle, and tonsillar engagement at the level of the foramen magnum. He underwent posterior fossa decompression and EVD revision at the same setting and remained in the intensive care unit for 10 days. He then underwent insertion of a ventriculoperitoneal (VP) shunt (medium pressure valve) as weaning from EVD was unsuccessful. On postop day 9, the VP shunt was removed, and a new EVD was placed again as his general condition deteriorated with fever, and cerebro-spinal fluid (CSF) findings were consistent with bacterial meningitis. Intravenous (IV) colistin (5 mg/kg/day) and Trimethoprim-Sulfamethoxazole (TMP-SMX; 10 mg/kg trimethoprim) were added to the treatment regimen upon growth of multidrug-resistant *Acinetobacter baumannii* in the CSF culture. In the third week of the treatment, while still on antibiotherapy (Meropenem + Colistin) and after three negative CSF cultures, the patient underwent VP shunt placement again (this time, low pressure valve was implanted due to the development of low-pressure hydrocephalus). On postoperative day 3, *C. parapsilosis* growth was detected from the CSF and EVD catheter tip samples, which were sent intraoperatively. The patient was then started on IV liposomal amphotericin B (3 mg/kg/day) and IV fluconazole (2 x 400 mg loading dose, 1 x 400 mg maintenance dose). After 2 weeks of IV antifungal therapy, he was switched to peroral (PO) fluconazole (2 x 200 mg/day) and was discharged home on this medication once CSF sterility and clinical improvement were achieved.

The patient was then lost to follow-up for 6 months. He was then admitted to the emergency department again due to severe vomiting. His neurologic exam was E4M6VT (he had been followed up at another center due to pneumonia, where a tracheotomy had been performed and his shunt was revised). He appeared to have poor personal hygiene and nutritional status. His physical exam revealed a clear discharge from the distal catheter incision site in the right upper quadrant. A cystic formation at the distal end of the shunt tubing and air-fluid levels consistent with ileus were detected on abdominal imaging, and enlargement of ventricles on cranial CT. In addition, chest CT was consistent with pneumonia. On further questioning, we were informed that the patient had had an abdominal discharge from the wound for approximately 3 months without any overt sign or symptom of meningitis. Ultra-sonography (USG)-guided percutaneous sampling from the abdominal cyst revealed yeast and pseudohyphae. He then underwent removal of his VP shunt, and an EVD was placed.

**Figure 1:**
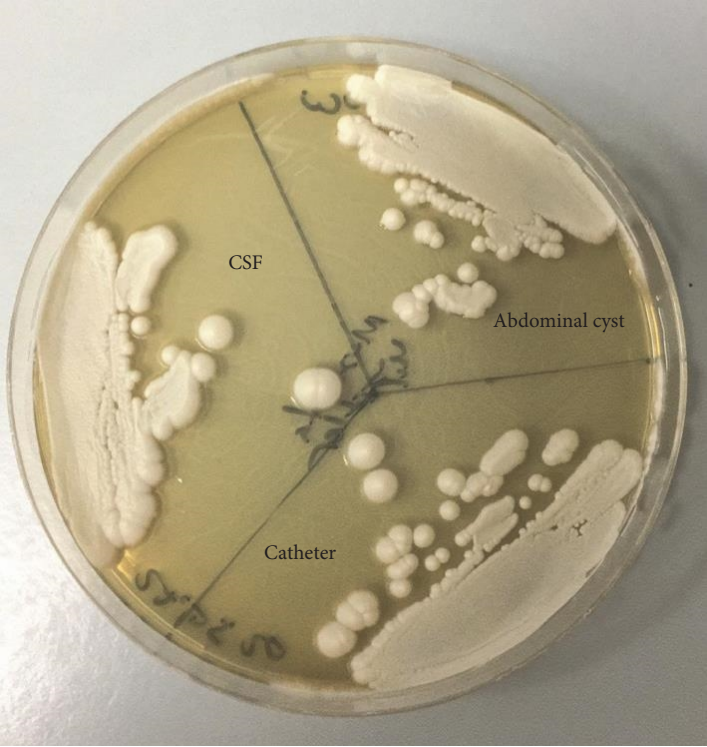
White, creamy, shiny, and smooth/creased colonies in Saburaud Dextrose Agar.

**Figure 2:**
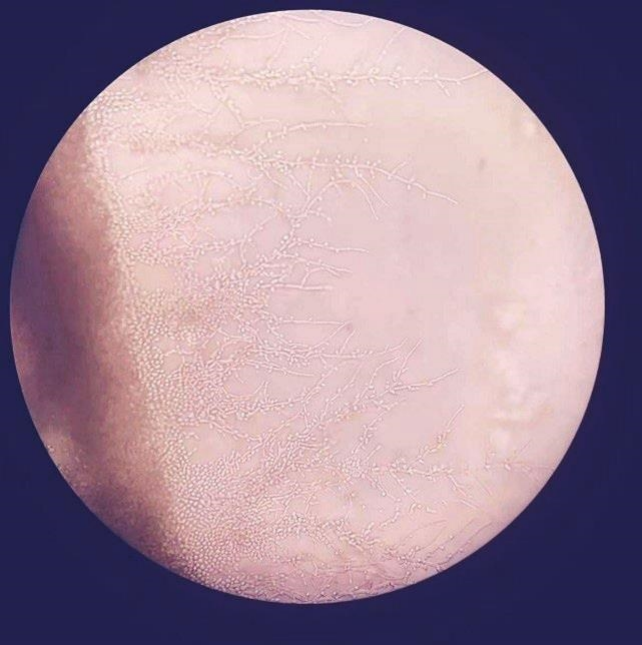
Morphologic appearance in corn meal agar with Tween 80, 10x magnification. Blastoconidia clusters, pseudohyphae in different directions, and characteristic large and curved pseudohyphae that are usually defined as “giant cells” of *C.parapsilosis*.

Yeast and pseudohyphae were also observed in Gram staining of the VP shunt catheter and CSF. The samples taken intraoperatively were examined at the mycology laboratory, where they were incubated at 37 °C for 72 h later at Sabouraud Dextrose Agar, chromogenic agar, and corn meal agar with Tween 80 at 25 °C. Consequently, *C. parapsilosis* growth was detected (Figures 1 and 2).

Based on these findings, the patient was started on IV liposomal amphotericin B and fluconazole. His ileus was managed conservatively. IV and intrathecal (IT) colistin and IV meropenem treatment were added due to *Acinetobacter baumanii* growth in blood cultures. The patient's clinical condition and inflammatory parameters did not improve under multiple antibiotherapy, and after having developed multiorgan failure, he expired on the postoperative 28thday (Tables 1, 2, and 3).

## 3. Discussion

Infection is still a dreaded complication of VP shunt surgery and may lead to significant morbidity and mortality [3]. In this context, fungal culprits are rare. Candida species account for about 1% of shunt infections [4], *Candida albicans* being the most common [5]. While it usually leads to an altered level of consciousness, other typical signs of infection, such as fever and neck stiffness, are relatively rare (10%) [1, 6]. The clinical course can also be further complicated by the coexistence of an abdominal pseudocyst [7].

*C. parapsilosis* is typically found in human skin flora. It is also the most commonly isolated yeast type in the hands of health care workers. *C. parapsilosis* is known to have the potential to grow through forming a biofilm on catheters and other medical implants [8]. There has been an increasing trend in the incidence of *C. parapsilosis*, which usually presents with endocarditis, peritonitis, sepsis, and ocular infection [9]. Central nervous system (CNS) infections and VP shunt infections due to this pathogen are still exceedingly rare even in patients who have a history of long-term antibiotic use, immune-compromised newborns, and intensive care unit patients [8]. Reported cases were mostly premature newborns with permanent catheters [9]. In a study by Yapar et al. [10], *C. parapsilosis* was detected in 21% of patients with candidemia, in ICU patients second to *C. albicans* (53%). They reported tracheotomy, femoral artery catheterization, RBC transfusion, TPN, abdominal surgery, and previous use of antibiotics as risk factors in cases with candidemia.

In our patient, long-term antibiotic (including perioperative prophylaxis) and corticosteroid use, history of multiple episodes of ICU stay, and multiple cranial surgeries were the potential predisposing factors for a fungal shunt infection (Tables 1, 2, and 3). One can certainly add the history of clear discharge from the right upper quadrant for 3 months to this list. There have been a handful of reports on *C. parapsilosis*-related VP shunt infection in newborns and children, but to the best of our knowledge, only two cases were reported in adults.

In the case reported by Bagheri et al. [9], their patient had undergone VP shunt surgery and developed mental status change during follow-up. Yeast growth was detected in CSF culture. The patient was treated with caspofungin, amphotericin B, flucytosine, and fluconazole. The shunt was removed, and EVD was placed on the 10^th^ day as mental status did not improve, and severe sepsis developed. Subsequently, infection parameters improved and culture negativity was obtained. After a 3-month follow-up, clinical improvement was observed.

The case reported by Fadel et al. [11] is quite similar to ours. In this case, the patient underwent posterior fossa decompression and VP shunt due to traumatic hemorrhage, and drainage from the abdominal wound site developed 8 months after discharge. Radiological examination revealed a pseudocyst in the abdomen. The patient had a clear drainage for about 2 months without the presence of any neurological symptoms. *C. parapsilosis* growth was detected in the CSF, the shunt was removed, and an EVD was inserted. Treatment with amphotericin B was started and had to be substituted with fluconazole due to amphotericin B-associated nephrotoxicity. VP shunt was put back in as his clinical condition improved, and CSF cultures were negative following 3 weeks of fluconazole treatment.

Despite the absence of a consensus regarding management of invasive *C. parapsilosis* infection, the sensible goals would be source control along with systemic antifungal treatment [1, 8]. Echinocandins have become the preferred treatment for most cases of candidemia and invasive candidiasis, except for infections involving the CNS, eyes, and urinary tract [12]. Additionally, *C. parapsilosis* exhibits unique intrinsic resistance to echinocandins. While patients with systemic infections often respond well to echinocandin therapy, even with high MIC values, repeated exposure to these drugs poses a risk for the development of resistance in *C. parapsilosis* [13]. The recent clinical guidelines for management of candida infections of the CNS recommend shunt removal as the initial step and administration of IV liposomal amphotericin B (3–5 mg/kg/day) and PO flucytosine (25 mg/kg/dose qid) (or IV liposomal amphotericin B alone). IV or PO fluconazole treatment (400–800 mg/day) is the most common alternative due to the nephrotoxicity of liposomal amphotericin B. Fluconazole is also effective for the treatment of *C. parapsilosis* and other candidal CNS infections [12].

In our case, IV liposomal amphotericin B (3 mg/kg/day) and IV fluconazole (400 mg/day maintenance dose following 800 mg loading dose) were administered. Antifungal treatment is recommended to continue until signs and symptoms improve and CSF studies return to normal [12].

The 6-month gap in patient follow-up is significant, as it likely contributed to the severe progression of the disease. Studies indicate that most shunt revisions occur within the first 6 months following the initial shunt operation [14], and subsequent revisions increase the risk of further shunt infections in patients [15]. Our patient demonstrated poor personal hygiene and nutritional status and had an abdominal discharge from the wound for approximately 3 months, without any obvious signs or symptoms of meningitis. In the postoperative period, patients at risk for revision and those in poor condition should be followed up more frequently and meticulously. Routine clinic visits continue to play a crucial role in identifying patients who may require shunt revision surgery and alter clinical care accordingly.

## 4. Conclusion

Since *C. parapsilosis*-related shunt infections are exceedingly rare in adults, a well-established specific treatment algorithm is not available. However, based on the knowledge from the pediatric population and other fungal infections, removal of the infected shunt along with the use of antifungal agents appears to be the two main steps of treatment [16, 17]. In our case, the patient developed multiorgan failure and expired despite the fact that this approach was taken. Lack of proper follow-up, having undergone VP shunt surgery in another facility, and a history of sustained discharge from the abdominal incision site following this surgery, along with poor personal hygiene and nutritional status, constituted additional risk factors for this course and limited our control in the management of this patient.

## Figures and Tables

**Table 1:** Clinical course in choronological order (First admission, ICU course).

Time course (Days)	Procedures	CSF growth	Antimicrobial agent
0	EVD insertion	—	IV ampicillin/sulbactam*⁣*^*∗*^
0	Endovascular embolization	—	IV ampicillin/sulbactam*⁣*^*∗*^
3	Posterior fossa decompression + EVD revision	—	IV ampicillin/sulbactam*⁣*^*∗*^
23	EVD removal	—	—

*Note:⁣* ^*∗*^Prophylaxis

**Table 2:** Clinical course in choronological order (First admission, progression of hydrocephalus 3 weeks following removal of the last EVD).

Time course (Days)	Procedures	CSF growth	Antimicrobial agent
0	EVD insertion	—	IV cefazolin*⁣*^*∗*^
2	VP shunt insertion	—	IV cefazolin + vancomycin*⁣*^*∗*^
11	Lumbar puncture	*A. baumannii*	IV colistin + meropenem + TMP-SMX
12	VP shunt removal + EVD insertion	*A. baumannii*	IV colistin + meropenem + TMP-SMX
18	EVD revision	—	IV colistin + meropenem TMP-SMX
25	VP shunt insertion	*C. parapsilosis*	IV colistin + meropenem (IV fluconazole and amphotericin B started after mycotic growth)
60	Consecutive CSF culture negativity		D/C home on PO fluconazole

*Note*: *⁣*^*∗*^Prophylaxis

**Table 3:** Clinical course in choronological order (Second admission).

Time course (Days)	Procedures	CSF growth	Antimicrobial agent
0	VP shunt removal	*C. parapsilosis*	IV fluconazole + amphotericin B + colistin (IV and IT)
12	EVD revision	—	IV fluconazole + amphotericin B + colistin (IV and IT)
24	EVD revision	—	IV fluconazole + amphotericin B + colistin (IV and IT)
28	EXITUS		

## Data Availability

The data supporting the findings of this study are available from the corresponding author upon reasonable request.

## Nomenclature

CNS: Central nervous system
CSF: Cerebro-spinal fluid
CT: Computed tomography
EVD: External ventricular drain
IT: Intrathecal
IV: Intravenous
LP: Lumbar puncture
PO: Peroral
TMP-SMX: Trimethoprim/sulfamethoxazole
USG: Ultra-sonography
VP: Ventriculoperitoneal.
